# Prognostic biomarker tumor-infiltrating lymphocytes failed to serve as a predictive biomarker for postoperative radiotherapy in completely resected pN2 non-small cell lung cancer: a retrospective analysis

**DOI:** 10.1186/s12931-024-02863-6

**Published:** 2024-06-17

**Authors:** Jiaran Li, Li Li, Jingjing Wang, Ning Liu, Haixin Liu, Fuhao Xu, Mengke Li, Shuanghu Yuan

**Affiliations:** 1 Shandong University Cancer Center, Jinan, Shandong China; 2 Department of Radiation Oncology, Shandong Provincial Key Laboratory of Radiation Oncology, Shandong Cancer Hospital and Institute, Shandong First Medical University, Shandong Academy of Medical Sciences, Jinan, Shandong China; 3 Department of Pathology, Shandong Cancer Hospital and Institute, Shandong First Medical University, Shandong Academy of Medical Sciences, Jinan, Shandong China; 4 Department of Oncology, Qingdao Municipal Hospital (Group), Qingdao, Shandong China; 5 Department of Radiation Oncology, Division of Life Sciences and Medicine, The First Affiliated Hospital of USTC, University of Science and Technology of China, No.17 Lujiang Road, Hefei, Anhui 230001 China; 6 Department of Radiation Oncology, Anhui Provincial Cancer Hospital, Hefei, Anhui China

**Keywords:** Tumor-infiltrating lymphocytes, Postoperative radiotherapy, Non-small cell lung cancer, Pathologic N2

## Abstract

**Background:**

Evidence suggests that radiotherapy is a potent immunomodulator in non-small cell lung cancer (NSCLC). Conversely, it has rarely been demonstrated if immune infiltration can influence radiotherapy efficacy. Herein, we explored the effect of tumor-infiltrating lymphocytes (TILs) on the response to postoperative radiotherapy (PORT) in completely resected stage III-pN2 NSCLC.

**Methods:**

This retrospective study included 244 patients with pathologically confirmed stage III-N2 NSCLC who underwent complete resection at our institution between 2014 and 2020. TILs were assessed with permanent full-face hematoxylin and eosin (H&E) sections and the evaluation of TILs was based on a published guideline. Patients were stratified into the TIL^low^ or TIL^high^ group with a cutoff value of 50%. Kaplan-Meier method and Log‐rank test were utilized to assess disease-free survival (DFS) and overall survival (OS). Univariate and multivariate Cox regression analysis were conducted to determine prognostic indicators.

**Results:**

Among 244 patients, a total of 121 patients received PORT whereas 123 did not. TILs level in patients with PORT was significantly higher than that in patients without PORT (*p* < 0.001). High TILs level was significantly associated with an improved DFS and OS in all the entire chort (DFS, *p* < 0.001; OS, *p* = 0.001), PORT chort (DFS, *p* = 0.003; OS, *p* = 0.011) and non-PORT chort (DFS, *p* < 0.001; OS, *p* = 0.034). There were no significant survival differences between different treatment modalities in the low TILs infiltration (DFS, *p* = 0.244; OS, *p* = 0.404) and high TILs infiltration (DFS, *p* = 0.167; OS, *p* = 0.958) groups.

**Conclusions:**

TILs evaluated with H&E sections could represent a prognostic biomarker in patients with completely resected pN2 NSCLC, and high TILs infiltration was associated with favorable survival outcomes.The predictive value of TILs for PORT still need to be further explored in the future.

## Introduction

Lung cancer is the most common cause of cancer-related death worldwide, with an estimated 1.8 million deaths each year [1]. Non-small cell lung cancer (NSCLC) is the major pathological type of lung cancer, consisting of approximately 85% of all lung cancer cases [2]. Among the various stages, Stage III NSCLC with N2 mediastinal nodal involvement, represents a heterogeneous patient population who require variable and individualized therapeutic approaches, and surgical resection is the primary treatment modality for these patients [3]. But there is still a notable risk of local–regional recurrence and distant metastasis in these patients who receive surgical resection alone [4].

For many years, postoperative radiotherapy (PORT) in completely resected pN2 NSCLC has been a subject of debate. Recently published PORT-C and Lung ART trials both concluded that PORT cannot be routinely recommended for pN2 NSCLC patients, posing a growing challenge to the acceptance of PORT in this context [5, 6]. Despite this, PORT has shown promising results in improving local control and overall survival (OS) rates in patients with high N2 mediastinal nodal burden disease [7–10].

The tumor-infiltrating lymphocytes (TILs), which is the major component of the tumor microenvironment (TME), was found to be significantly associated with enhanced antitumor activity and tumor destruction [11]. Several studies have demonstrated that the presence and density of TILs within the tumor tissue can serve as predictive and prognostic biomarkers of the treatment response and overall prognosis in NSCLC patients [12, 13]. Radiotherapy, as an important means for local treatment, can convert an immunologically silenced “cold” tumor to an active “hot” tumor by triggering the release of pro-inflammatory mediators and increasing tumor-infiltrating immune cells [14]. However, an important question remains unanswered: could TILs be used as a predictive biomarker to predict the efficacy of radiotherapy?

Hence, the present study aims to investigate the utility of TILs evaluated through H&E sections as biomarkers for predicting PORT efficacy in patients with completely resected stage III pN2 NSCLC and futher identify the benefit population for PORT from the perspective of immune infiltration.

## Materials and methods

### Patient cohort

**Fig. 1:**
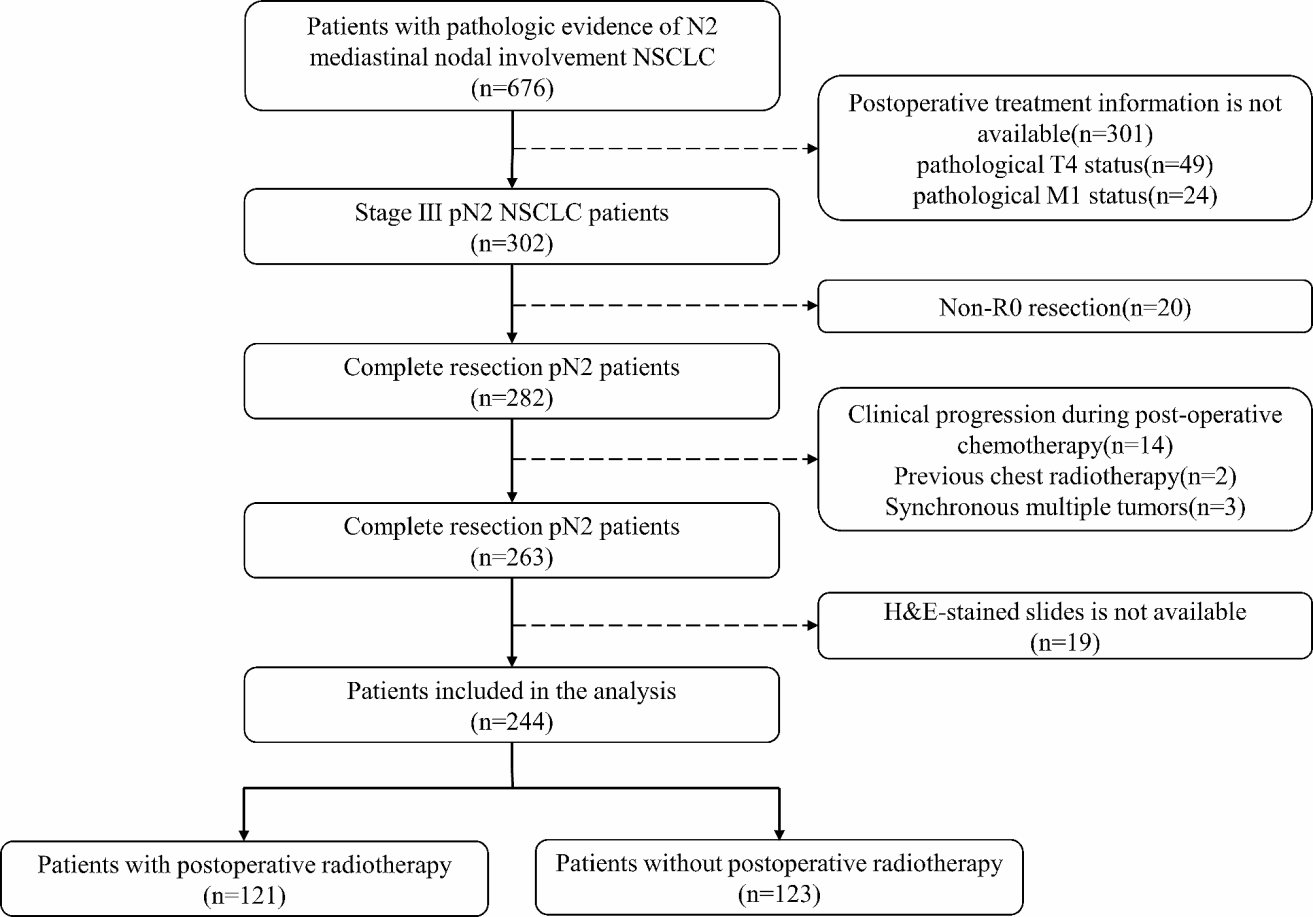
Recruitment and selection process of patients

This retrospective study included patients with stage III pN2 NSCLC who was histologically documented treated by surgery combined with chemotherapy ± radiotherapy in Shandong Cancer Hospital and Institute between 2014 and 2020. The inclusion criteria were (1) pathologic evidence of NSCLC and R0 resection was achieved; (2) pathologically documented N2 mediastinal nodal involvement at the time of surgery; (3) receiving 2–4 cycles of platinum-based doublet adjuvant chemotherapy following surgery, neoadjuvant chemotherapy was also allowed; (4) three-dimensional conformal radiation therapy or intensity-modulated radiation therapy was administered at a dose of 50 ~ 60 Gy; (5) available clinicopathologic data and H&E sections. The exclusion criteria were (1) pathological T4 and M1 status; (2) locoregional recurrence or distant metastasis during postoperative chemotherapy; (3) previous chest radiotherapy; (4) synchronous multiple primary cancers. Ultimately, a total of 244 patients were included in this study. The patient recruitment and selection process was shown in Fig. 1. The study was approved by the ethical review committee of our hospital (ethics approval number: SDTHEC2020004042).

### Treatment response assessment

The follow-up of all patients was conducted every 3 months during the first 2 years after surgery. After that, the patients were followed up every 6 ~ 12 months. Treatment responses were assessed by CT imaging at each follow-up and compared to the images at baseline or from the last follow-up and were evaluated according to RECIST 1.1. Disease-free survival (DFS) was defined as the time from the surgery date to the date of first locoregional recurrence, distant metastasis or death of any cause. OS was defined as the time from surgery to the death of the patient or the last follow-up. All time-to-event data were censored at last follow-up if the corresponding event had not occurred.

### Histological evaluation

Permanent full-face H&E sections from surgical specimens from each case were retrieved from the pathology archives and evaluated for TILs. TILs were assessed by two experienced pathologists who were blinded to each other, and the evaluation of TILs was primarily based on the guidelines from International Immuno-Oncology Biomarkers Working Group [15]. We assessed TILs level within the areas including invasive margin and the stromal compartment within the tumor border. Only mononuclear cells (lymphocytes and plasma cells) were included in the assessment. Areas with necrosis, crush artifacts, and fibrosis were excluded for TILs evaluation. The level of TILs was scored as a continuous variable according to the percentage of stroma area occupied by stromal TILs and was evaluated as a percentage in 5% increments. The percentage assessments for multiple slides from each case were averaged. And then patients were subdivided into TIL^low^ group (≤50%) and TIL^high^ group (>50%) according to arbitrary TILs level of 50%.

### Statistical analysis

Patients’ clinicopathological characteristics were described for the whole cohort and compared using the Chi-squared or Fisher exact test, as appropriate. The Mann–Whitney test was used for the comparison of TILs level between patients with and without PORT. OS and DFS were described by the Kaplan–Meier (KM) curves, and the log-rank test was used to compare whether there was a survival difference between two groups. Median follow-up was estimated with the reverse KM method [16]. Cox proportional hazard model was used for univariate and multivariate analyses. All statistical analyses were conducted using SPSS version 25.0 software (IBM, USA). The optimal cutoff value of TILs was determined by the extension module Survival Module of ClinicoPath in Jamovi software (version 2.3.21). All graphs were made using GraphPad software (version 9.5.1). The factors with *p* < 0.10 on univariate analysis were included in the multivariate analysis, and *p* < 0.05 was considered to be statistically significant. All tests were two-tailed.

## Results

### Baseline characteristics

**Fig. 2:**
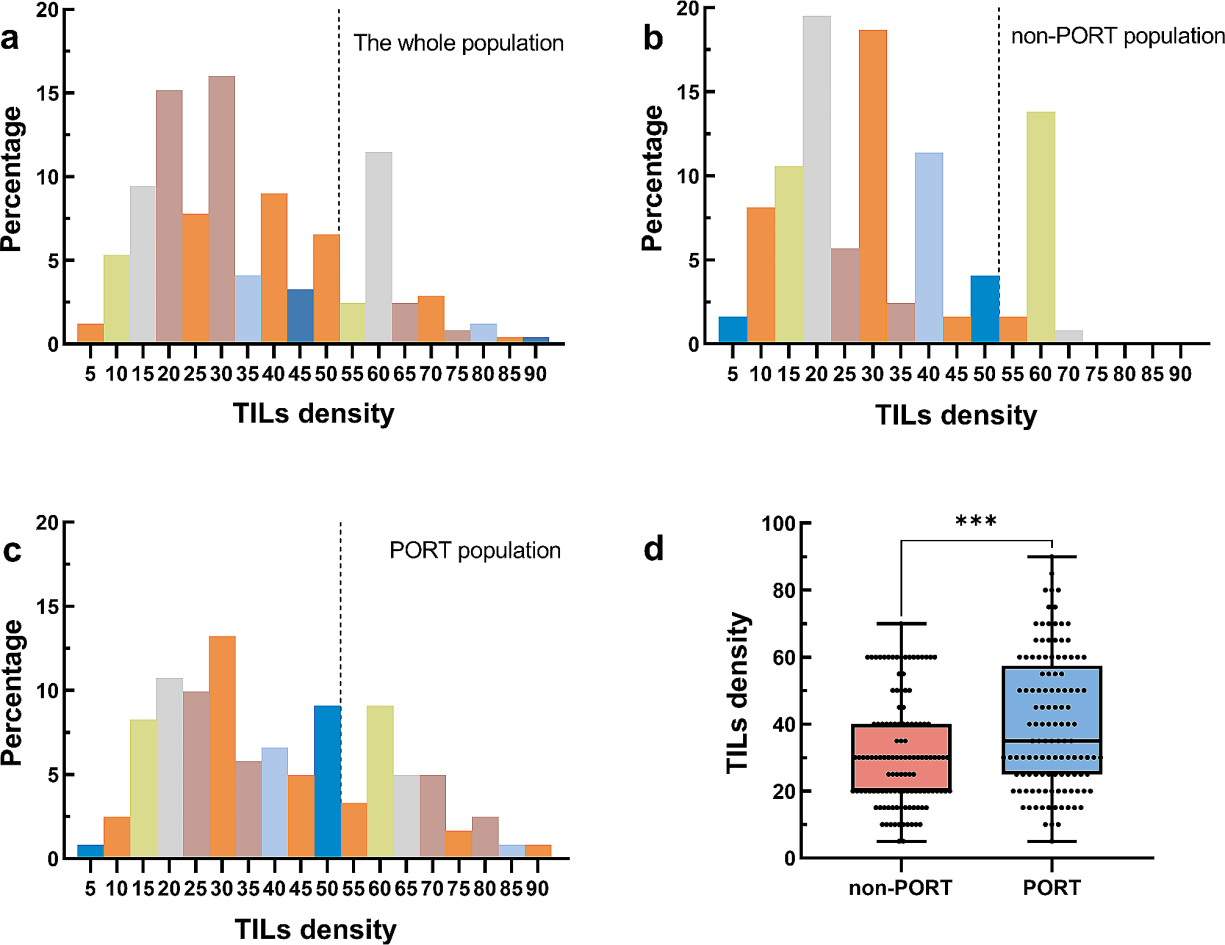
Distribution of tumor-infiltrating lymphocytes (TILs) within the (**a**) whole population, (**b**) non-PORT population and (**c**) PORT population and (**d**) the differences of TILs between patients with and without PORT . The dashed black line indicates cut-off value

**Fig. 3:**
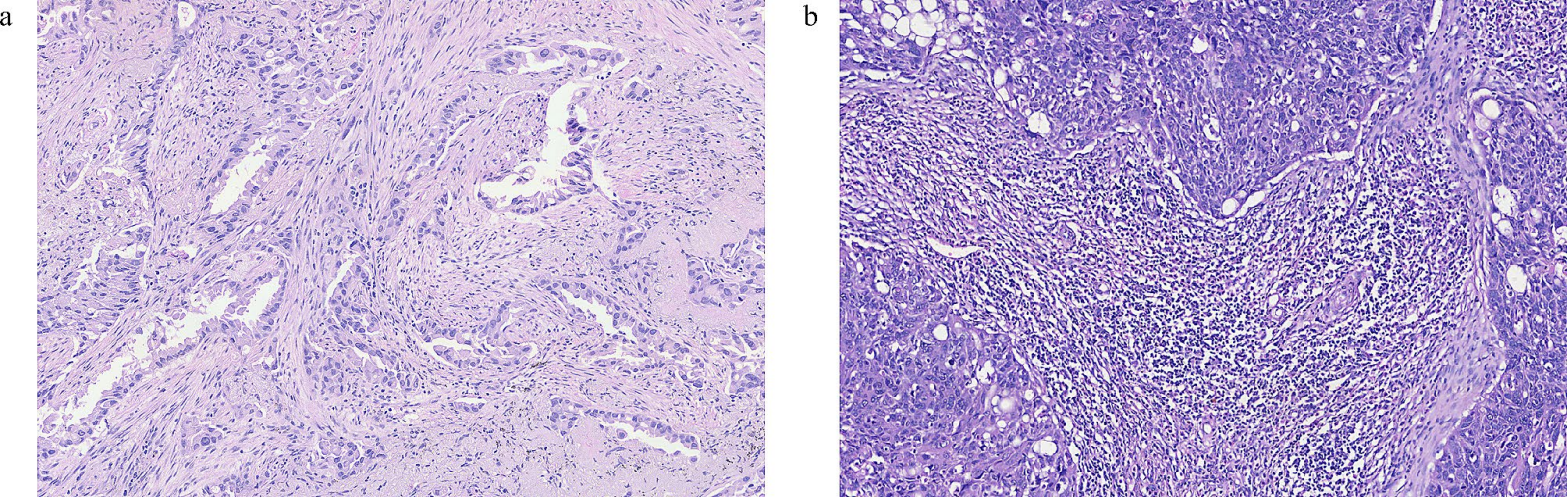
Example of stromal tumor-infiltrating lymphocytes (TILs) assessment with hematoxylin and eosin (H&E) sections. (**a**) Low and **(b)** high level of TILs in NSCLC tissue

We retrospectively performed analyses on 244 stage III pN2 patients who met the inclusion criteria in the present study, and patients’ clinicopathological characteristics were shown in Table 1. The distribution of TILs was illustrated in Fig. 2. TILs level in patients with PORT was significantly higher than that in patients without PORT (Fig. 2d). For further analyses the cohort was divided into two groups based on TILs density, of whom 190 patients (77.9%) were assigned to TIL^low^ group and 54 patients (22.1%) were TIL^high^ group. Representative images of two groups were provided in Fig. 3.

**Table 1:** Patients clinicopathological characteristics of the whole cohort

Characteristics	Total	non-PORT	PORT	*p* value
	(*n* = 244)	(*n* = 123)	(*n* = 121)	
Gender				0.867
Male	154 (63.1)	77 (62.6)	77 (63.6)	
Female	90 (36.9)	46 (37.4)	44 (36.4)	
Age				0.977
≤60	149 (61.1)	75 (61.0)	74 (61.2)	
> 60	95 (38.9)	48 (39.0)	47 (38.8)	
Tobacco				0.516
Absence	139 (57.4)	72 (59.5)	67 (55.4)	
Presence	103 (42.6)	49 (40.5)	54 (44.6)	
Tumor Side				0.503
Left	102 (41.8)	54 (43.9)	48 (39.7)	
Right	142 (58.2)	69 (56.1)	73 (60.3)	
Histology				0.482
SCC	58 (23.8)	33 (26.8)	25 (20.7)	
ADC	175 (71.7)	84 (68.3)	91 (75.2)	
Others	11 (4.5)	6 (4.9)	5 (4.1)	
Differentiation				0.427
Well	17 (7.0)	6 (4.9)	11 (9.1)	
Moderate	154 (63.4)	80 (65.6)	74 (61.2)	
Poor	72 (29.6)	36 (29.5)	36 (29.8)	
Vascular invasion				0.183
No	220 (90.2)	114 (92.7)	106 (87.6)	
Yes	24 (9.8)	9 (7.3)	15 (12.4)	
TIL level				0.026
Low	190 (77.9)	103 (83.7)	87 (71.9)	
High	54 (22.1)	20 (16.3)	34 (28.1)	
pT Stage				0.877
T1	68 (27.9)	34 (27.6)	34 (28.1)	
T2	143 (58.6)	71 (57.7)	72 (59.5)	
T3	33 (13.5)	18 (14.6)	15 (12.4)	
Involved N2 stations				0.620
Single	149 (61.1)	77 (62.6)	72 (59.5)	
Multiple	95 (38.9)	46 (37.4)	49 (40.5)	
LNR				0.684
< 50%	193 (79.1)	96 (78.0)	97 (80.2)	
≥50%	51 (20.9)	27 (22.0)	24 (19.8)	
Highest LNs				0.559
Negative	83 (34.0)	44 (35.8)	39 (32.2)	
Positive	161 (66.0)	79 (64.2)	82 (67.8)	
Neoadjuvant Chemotherapy				0.211
No	204 (84.0)	106 (86.9)	98 (81.0)	
Yes	39 (16.0)	16 (13.1)	23 (19.0)	
Type of surgery				0.030
Sublobar resection	9 (3.7)	1 (0.8)	8 (6.6)	
Lobectomy	195 (79.9)	98 (79.7)	97 (80.2)	
Bilobectomy	40 (16.4)	24 (19.5)	16 (13.2)	

Abbreviations: TIL: Tumor-infiltrating lymphocytes, SCC: squamous cell carcinoma, ADC: adenocarcinoma, LNR: lymph node ratio, LNs: lymph nodes, PORT: postoperative radiotherapy

### Associations between TILs and prognosis

At final data cut-off, the median follow-up time was 44.6 months. During the follow-up period, there were 159 DFS events (65.2%) and 65 OS events (26.6%) in the entire cohort. Median DFS and OS were 26.5 and 95.5 months, respectively.

**Fig. 4:**
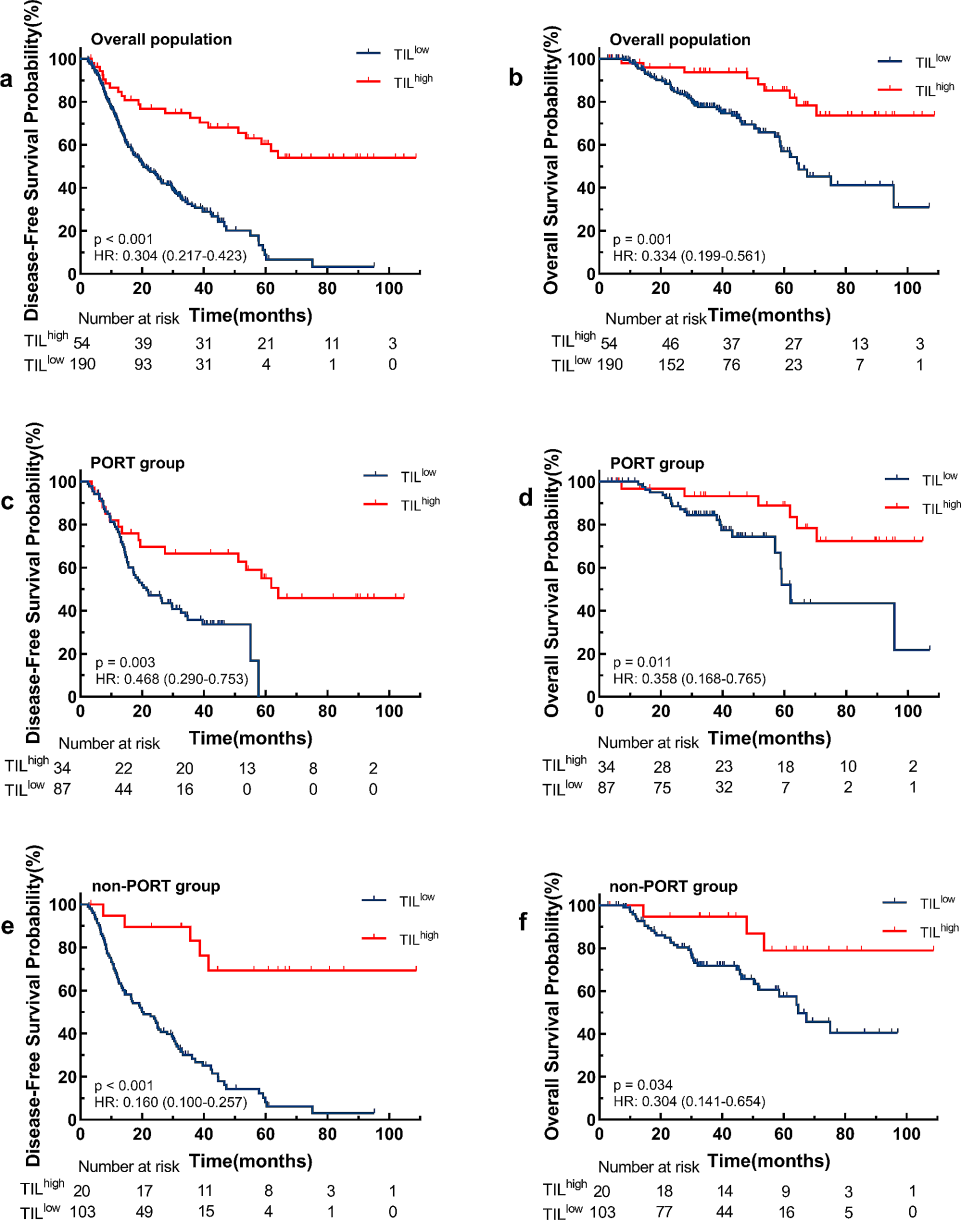
Kaplan-Meier estimates of DFS and OS for patients with high and low tumor-infiltrating lymphocytes (TILs) level within the whole population, PORT population and non-PORT population

We found that TIL^high^ group was associated with an improved prognosis for the entire chort. The TIL^high^ group had longer OS than the TIL^low^ group [hazard ratio (HR) 0.334, 95% CI 0.199−0.561, *p* = 0.001] (Fig. 4a). The median OS was 64.7 months in the TIL^low^ group but not reached in the TIL^high^ group. Similar results were observed for DFS (HR 0.304, 95% CI 0.217−0.423, *p* < 0.001) (Fig. 4b). The median DFS time in the TIL^low^ group was 20.5 months, while the median DFS time in the TIL^high^ group was also not reached. In multivariable analysis, high TILs level remained a good predictive factor for both OS (HR 0.589, 95% CI 0.356−0.975, *p* = 0.007) (Table 2) and DFS (HR 0.290, 95% CI 0.174−0.483, *p* < 0.001) (Table 3) after correcting for factors which were significant in univariate analysis. Notworthy, the significant positive correlation between high TILs infiltration and improved survival outcomes was observed in both PORT chort (Fig. 4c **and d**) and non-PORT chort (Fig. 4e **and f**).

**Table 2:** Univariate and multivariate analyses of the OS in all patients

Characteristics	Univariate analysis		Multivariable analysis		
	HR (95%CI)	*p* value	HR (95%CI)	*p* value	
Gender		0.281			
Male	1				
Female	0.751 (0.446–1.264)				
Age		0.384			
≤60	1				
> 60	1.246 (0.855–4.028)				
Tobacco		0.800			
Absence	1				
Presence	1.066 (0.649–1.752)				
Tumor Side		0.078		0.040	
Left	1		1		
Right	0.645 (0.395–1.051)		0.589 (0.356–0.975)		
TIL level		0.002		0.007	
TIL^low^	1		1		
TIL^high^	0.313 (0.152–0.642)		0.589 (0.356–0.975)		
Histology		0.622			
SCC	1				
ADC	0.730 (0.425–1.256)				
Others	1.792 (0.526-6.100)				
Differentiation		0.542			
Well	1				
Moderate	1.070 (0.419–2.734)				
Poor	1.251 (0.470–3.329)				
Vascular invasion		0.437			
No	1				
Yes	1.369 (0.620–3.023)				
pT Stage		0.083		0.060	
T1	1		1		
T2	1.762 (0.882–3.523)		1.749 (0.870–3.516)		
T3	2.072 (0.885–4.851)		2.248 (0.948–5.332)		
Involved N2 stations		<0.001		0.022	
Single	1		1		
Multiple	2.463 (1.503–4.038)		1.874 (1.094–3.209)		
LNR		0.001		0.164	
< 50%	1		1		
≥50%	2.351 (1.405–3.936)		1.494 (0.848–2.631)		
Highest LNs		0.555			
Negative	1				
Positive	1.172 (0.692–1.987)				
Neoadjuvant Chemotherapy		0.422			
No	1				
Yes	1.305 (0.681-2.500)				
PORT		0.155			
No	1				
Yes	0.699 (0.426–1.145)				
Type of surgery		0.001		0.004	
Sublobar resection	1		1		
Lobectomy	1.476 (0.356–6.126)		1.226 (0.292–5.149)		
Bilobectomy	3.740 (0.861–16.246)		2.917 (0.662–12.857)		

Abbreviations: TIL: Tumor-infiltrating lymphocytes, SCC: squamous cell carcinoma, ADC: adenocarcinoma, LNR: lymph node ratio, LNs: lymph nodes, PORT: postoperative radiotherapy

**Table 3:** Univariate and multivariate analyses of the DFS in all patients

**Characteristics**	Univariate analysis		Multivariable analysis	
	HR (95%CI)	p value	HR (95%CI)	p value
Gender		0.703		
Male	1			
Female	0.940 (0.682–1.294)			
Age		0.131		
≤60	1			
> 60	0.780 (0.565–1.077)			
Tobacco		0.910		
Absence	1			
Presence	0.982 (0.715–1.349)			
Tumor Side		0.165		
Left	1			
Right	0.801 (0.586–1.095)			
TIL level		<0.001		<0.001
TIL^low^	1		1	
TIL^high^	0.254 (0.155–0.416)		0.290 (0.174–0.483)	
Histology		0.022		0.146
SCC	1		1	
ADC	1.310 (0.884–1.942)		0.822 (0.532–1.270)	
Others	2.951 (1.399–6.222)		9.460 (4.194–21.335)	
Differentiation		0.556		
Well	1			
Moderate	1.140 (0.611–2.126)			
Poor	0.560 (0.631–2.341)			
Vascular invasion		0.188		
No	1			
Yes	1.393 (0.851–2.282)			
pT Stage		0.009		<0.001
T1	1		1	
T2	1.597 (1.075–2.372)		1.836 (1.221–2.760)	
T3	1.890 (1.118–3.193)		2.486 (1.439–4.293)	
Involved N2 stations		< 0.001		0.042
Single	1		1	
Multiple	1.804 (1.316–2.472)		1.427 (1.012–2.012)	
LNR		<0.001		0.319
< 50%	1		1	
≥50%	1.963 (1.387–2.779)		1.217 (0.827–1.791)	
Highest LNs		0.439		
Negative	1			
Positive	1.142 (0.815-1.600)			
Neoadjuvant Chemotherapy		0.005		0.389
No	1		1	
Yes	1.775 (1.184–2.601)		1.202 (0.791–1.824)	
PORT		0.111		
No	1			
Yes	0.775 (0.567–1.060)			
Type of surgery		0.025		0.168
Sublobar resection	1		1	
Lobectomy	0.716 (0.334–1.536)		0.674 (0.311–1.459)	
Bilobectomy	1.324 (0.582–3.012)		1.046 (0.455–2.404)	

Abbreviations: TIL: Tumor-infiltrating lymphocytes, SCC: squamous cell carcinoma, ADC: adenocarcinoma, LNR: lymph node ratio, LNs: lymph nodes, PORT: postoperative radiotherapy

#### Association between TILs and PORT Benefit

**Fig. 5:**
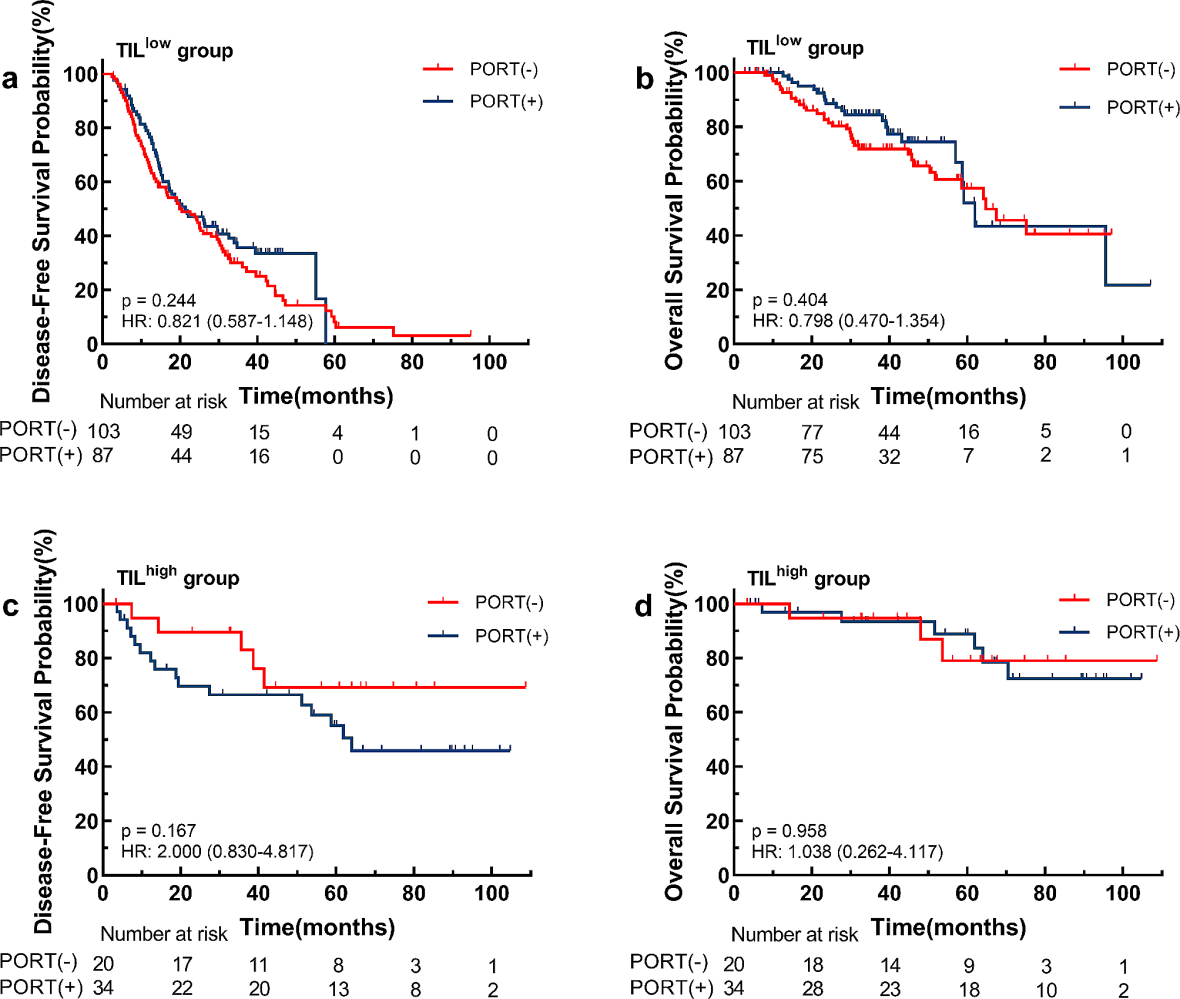
Kaplan–Meier curves comparing PORT versus non-PORT for two groups of patients with high and low tumor-infiltrating lymphocytes (TILs) level

Cox regression analysis showed that PORT was not an independent prognostic factor in patients with pN2 NSCLC (Tables 2 and 3). We further explored whether TIL infiltrations could predict PORT efficacy and better identify the population that will benefit from PORT. In the low TILs density group, patients treated with PORT had similar DFS (HR 0.821, 95% CI 0.587–1.148, *p* = 0.244) and OS (HR 0.798, 95% CI 0.470–1.354, *p* = 0.404) compared to patients without PORT **(**Fig. 5a **and b)**. Similar results were observed in the high TILs density group, there was no difference in DFS (HR 2.000, 95% CI 0.830–4.817, *p* = 0.167) and OS (HR 1.038, 95% CI 0.262–4.117, *p* = 0.958) related to treatment **(**Fig. 5c **and d)**.

## Discussion

In this study, we aimed to determine whether the TILs evaluated with H&E sections could predict the response to PORT in patients with completely resected pN2 NSCLC. Our results demonstrated that baseline TILs, an indicator of immunogenicity, could serve as a potent prognostic biomarker in pN2 patients, regardless of the treatment modality applied. The present study is the first study to investigate whether TILs can predict the efficacy of PORT. However, similar prognosis was achieved for patients treated with and without PORT in different TILs infiltration subgroups, and our study did not confirm the predictive value of TILs for the efficacy of PORT.

Immune cells play a critical role in the development and progression of cancer. Considering the important role of host immune infiltration in controlling tumor progression, immune infiltration could be served as a new prognostic factor in various tumor types [17]. It has been shown that higher level of TILs in the primary tumor site is associated with good prognosis with completely resected stage III-N2 NSCLC patients [13, 18]. Our results reached a consistent conclusion and coincided with those of previous reports. However, this study differed from previous studies in the following two ways. First, the sample sizes of patients treated with and without PORT enrolled in this study were similar. Second, our study further validated our results in different treatment subgroups.

The use of PORT in routine treatment settings for pN2 NSCLC had been a controversial issue since a meta-analysis in 1998 [19]. Recently, two large randomized clinical trials have both reported that PORT cannot be recommended in all stage III-N2 patients, but should identify the appropriate patients who will optimally benefit from PORT [5, 6]. Previous studies have shown that select patients could benefit from PORT from the perspective of N2 mediastinal nodal burden, such as a high lymph node ratio and multiple N2 stations [7–9]. Departing from previous research perspectives, we expect to identify the benefit population for PORT by utilizing TILs infiltration status, as a study has reported that TILs can be used as predictive markers to predict the efficacy of radiotherapy in breast cancer [20].

However, the present study failed to find the predictive value for the efficacy of PORT in completely resected pN2 NSCLC, as DFS and OS were not statistically different for patients receiving PORT and adjuvant chemotherapy in the subgroup analysis based on the TILs infiltration level. The results may be explained by the high cut-off value. The cut-off value of 50% in the present study was determined in the prognostic analysis, which is consistent with previous studies [21]. However, this cutoff value may not reflect the predictive value of the efficacy of radiotherapy. It was reported that stratification of TILs with a cutoff value of 10% could predict the risk for ipsilateral tumor recurrence in breat cancer research [20]. High TILs infiltration was a protective factor, reducing the risk of regional recurrence and distant recurrence. Thus, high cutoff value might underestimate the benefits of PORT and a lower cutoff value should be futher explored. Although the results were negative, our study fills a research gap in this field.

Our study still has some limitations. First, it is a retrospective study with a relatively small sample size, and the results might be flawed because of residual confounding factors. Second, it is based on single-institution data, and exploration in larger datasets is required to understand any differential efficacy of PORT based on TILs infiltration in patients with completely resected pN2 disease.

## Conclusions

Histologic assessment of TILs with H&E section had a strong prognostic value in patients with completely resected pN2 NSCLC. However, TILs had limited clinical utility in the setting of identifing the benefit population for PORT and futher studies shuold be should be conducted to investigate the predictive value of TILs for radiotherapy.

## Data Availability

The datasets used and analyzed during the current study are available from the corresponding author on reasonable request.

## Abbreviations

NSCLC: Non–small cell lung cancer
PORT: Postoperative radiotherapy
OS: Overall survival
TILs: Tumor-infiltrating lymphocytes
TME: Tumor microenvironment
IHC: Immunohistochemistry
H&E: Hematoxylin and eosin
DFS: Disease-free survival
KM: Kaplan–Meier
HR: Hazard ratio
